# Extending the Ring Theory of Personhood to the Care of Dying Patients in Intensive Care Units

**DOI:** 10.1007/s41649-021-00192-0

**Published:** 2021-10-20

**Authors:** Natalie Pei Xin Chan, Jeng Long Chia, Chong Yao Ho, Lisa Xin Ling Ngiam, Joshua Tze Yin Kuek, Nur Haidah Binte Ahmad Kamal, Ahmad Bin Hanifah Marican Abdurrahman, Yun Ting Ong, Min Chiam, Alexia Sze Inn Lee, Annelissa Mien Chew Chin, Stephen Mason, Lalit Kumar Radha Krishna

**Affiliations:** 1 Yong Loo Lin School of Medicine , National University of Singapore, Singapore; 2 Division of Supportive and Palliative Care , National Cancer Centre Singapore, Singapore; 3 Division of Cancer Education , National Cancer Centre Singapore, Singapore; 4 Medical Library, National University of Singapore Libraries, National University of Singapore, Singapore; 5 Palliative Care Institute Liverpool, Academic Palliative & End of Life Care Centre, University of Liverpool, Liverpool, UK; 6 Cancer Research Centre, University of Liverpool, Liverpool, UK; 7 Palliative Care Centre for Excellence in Research and Education (PalC), Singapore; 8 Centre for Biomedical Ethics, National University of Singapore , Singapore; 9 Duke-NUS Graduate Medical School, Singapore

**Keywords:** COVID-19, Intensive care unit (ICU), Death and dying, Personhood, Ring theory of personhood (RToP)

## Abstract

It is evident, in the face of the COVID-19 pandemic that has physicians confronting death and dying at unprecedented levels along with growing data suggesting that physicians who care for dying patients face complex emotional, psychological and behavioural effects, that there is a need for their better understanding and the implementation of supportive measures. Taking into account data positing that effects of caring for dying patients may impact a physician’s concept of personhood, or “what makes you, ‘you’”, we adopt Radha Krishna’s Ring Theory of Personhood (RToP) to scrutinise the experiences of physicians working in intensive care units (ICU) using a fictional scenario that was inspired by real events. The impact of death and dying, its catalysts, internal constituents, external factors, dyssynchrony, and buffers, specific to ICU physicians, were identified and explored. Such a framework allows for ramifications to be considered holistically and facilitates the curation of strategies for conflict resolution. This evaluation of the RToP acknowledges the experience and wide-ranging effects it has on ICU physicians. As such, our findings provide insight into their specific needs and highlight the importance of support on a personal and organisational level. Although further research needs to be conducted, the RToP could serve as the basis for a longitudinal assessment tool supported by the use of portfolios or mentorship due to their provision of personalised, appropriate, specific, timely, accessible and long-term support.

## Background

The COVID-19 pandemic has seen medical professionals taking to social media sites to share their knowledge and experiences with dying patients (Ouyang 2020a). Not only were these physicians educating their colleagues and the public on clinical developments surrounding the COVID-19 infections, they also provided rare insights into the human aspects of caring for the dying during this trying time (Engelhart and Smith 2020; Ouyang 2020b; Schmidt 2020). Perhaps nowhere has the impact of death and dying on physicians been as evident as in the intensive care unit (ICU) where stress, manpower shortages, evolving care conditions and complex treatment decisions (Barnett et al. 2016; Brooks et al. 2017b; Donnelly and Psirides 2015; Laurent et al. 2017; Pattison et al. 2013; Trankle 2014; Wåhlin et al. 2010)—such as limiting care for the aged, those with poor mobility, cancer and other life-limiting illnesses—have created emotional, psychological, moral and existential distress amongst physicians (Engelhart and Smith 2020; Ouyang 2020b; Schmidt 2020).

Indeed, so profound have these effects been that fundamental changes in the thoughts (Aslakson et al. 2010; Monteiro et al. 2016; Simmonds 1996; Svantesson et al. 2003), decision-making (Aslakson et al. 2010; Monteiro et al. 2016; Simmonds 1996; Svantesson et al. 2003) and relationships (Almansour et al. 2019; Amati and Hannawa 2014; Asch et al. 1995; Baggs et al. 2012; Brooks et al. 2017b; Chikhladze et al. 2016; Cohen et al. 2005; Gutierrez 2012; Hamric and Blackhall 2007; Hawryluck et al. 2002; Henrich et al. 2016; Hough et al. 2005; Laurent et al. 2017; Monteiro et al. 2016; Simmonds 1996; Trankle 2014; Wåhlin et al. 2010; Weng et al. 2011; Workman et al. 2003) of ICU physicians have been reported. In addition, how these physicians have come to view their professional responsibilities, and how they perceive the meeting of their professional duties and interactions, have evolved (Aslakson et al. 2010; Brooks et al. 2017a, 2017b; Donnelly and Psirides 2015; Festic et al. 2012; Hamric and Blackhall 2007; Jensen et al. 2013; Laurent et al. 2017; McAndrew and Leske 2015; Mehter et al. 2018; Nordgren and Olsson 2004; Robertsen et al. 2019; Svantesson et al. 2003; Trankle 2014; Wåhlin et al. 2010; Zambrano et al. 2012).

In this study, we use Radha Krishna’s definition of personhood, or “what makes you, you” (Radha Krishna et al. 2015; Radha Krishna and Alsuwaigh 2015, 1), from the Ring Theory of Personhood (RToP). With such wide ramifications reported, it might be said that the personhood of ICU physicians have also been altered (Daly et al. 2018; Henrich et al. 2016; Nordgren and Olsson 2004; Robertsen et al. 2019). The significance of such a hypothesis is vast. The notion that such experiences can shake and change the very foundations of a physician’s personhood (Begley 2020; Oliver 2020; Phua et al. 2020) suggests that addressing its effects will likely require a comprehensive approach. As a result, better understanding of these effects upon their personhood is required (Henrich et al. 2016).

## Current Understanding of Personhood

To discuss this issue holistically, we adopt Radha Krishna’s RToP (Radha Krishna et al. 2015; Radha Krishna and Alsuwaigh 2015), a clinically evidenced holistic concept of personhood created within the palliative care setting. Aside from being situated in the context of care for the dying, the RToP has also been shown to capture evolving concepts of personhood in changing conditions over time which would mirror anticipated changes in the ICU setting.

**Fig. 1:**
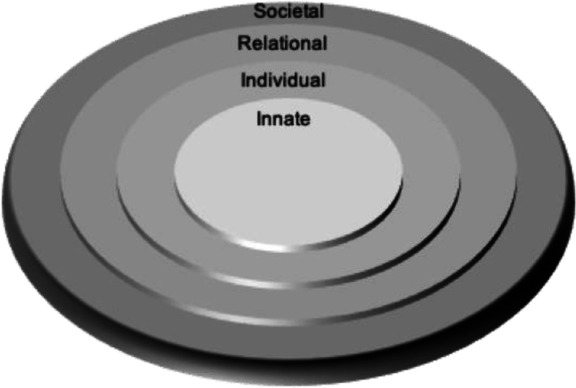
Ring Theory of Personhood. Radha Krishna’s Ring Theory of Personhood – personhood is conceived as four interconnected, concentric rings: innate, individual, relational and societal rings

Radha Krishna’s RToP conceives of personhood as four interconnected, concentric rings. These are referred to as the innate, individual, relational and societal rings (Fig. 1).

The *Innate Ring* comprises inalienable aspects of all humans. This status is conferred to each person by virtue of their connections with the divine or their genetic makeup which endows them with human features, for example their blood type which cannot be altered. The combination of all these considerations confers the individual respect, considerations and rights reserved for all human beings till death. The Innate Ring also considers aspects that a person is born into or with. These include their gender, ethnicity, facial features, skin and hair colour, family set up, familial, religious and cultural beliefs as well as their very culture and society. These features may be altered and are thus considered to be changeable aspects or the Secondary Innate Ring. By virtue of its association with religious and cultural belief systems, the Secondary Innate Ring contains beliefs, values and principles that the person abides by. As with all the rings, these influence thinking, preferences, decision-making, interactions and behaviours.

The *Individual Ring* is built upon the Innate Ring and relates to a person’s conscious functions. These include their ability for cogitation, communication, self-awareness and action. By virtue of their thoughts, preferences and experiences, there are also beliefs, values, motivations and principles rooted in the Individual Ring. These may overlap with those of the Innate Ring which are rooted in familial, cultural and religious beliefs highlighting the interconnectedness of the rings. The RToP’s ability to detect change in the Individual Ring will help to better understand the impact on ICU physicians’ thinking, emotions and coping (Dyregrov and Gjestad 2011; Youngblut et al. 2017).

The *Relational Ring* comprises of close personal relationships as defined by the individual, and mutually reciprocated by the other party. There are some relationships that are apparent from birth, such as those with parents and siblings, conferred by virtue of being born into a family. There are other relationships that develop through interaction, such as those formed with close friends. What makes a relationship important, how individuals within this ring are to behave and how they are to be treated by the person creates specific beliefs, values and principles housed in this ring.

The *Societal Ring* contains two domains. One, relationships not deemed to be significant by the person will be placed in their Relational Ring. Peripheral relationships that fall into this category include acquaintances, co-workers and may even include family members the individual may not be as intimate with. Also associated with this domain are the specific societal beliefs, values and principles that determine how individuals within this ring are to behave and how they are to be treated by the person. Two, the Societal Ring contains enculturated roles, responsibilities and expectations that the person is bound to by virtue of their being a part of a specific society. It obliges the person to comply with legal, ethical, sociocultural and societal standards and also confers them their basic rights within the society. The size of this ring is dependent on the number of relationships contained within the ring, and its depth is dependent upon the amount of influence the societal element has over the other rings. For physicians, for example, the Societal Ring establishes acceptable standards of practice, desirable work ethic and satisfactory codes of conduct, and captures society’s support and considerations for ICU physicians.

Perhaps more significantly, each ring contains specific values, beliefs, principles and expectations that come together within the Individual Ring and influence preferences, motivations, decisions and biases, thoughts, and actions. This highlights the interrelatedness of the rings and the central role of the Individual Ring. Concurrently, changing conditions (Barr and Cacciatore 2008), evolving contextual (Wei et al. 2016), existential, personal, relational and societal considerations also impact the individual’s thoughts and actions. This underlines the importance of the RToP’s ability to capture changes in thinking, coping (Dyregrov and Gjestad 2011; Youngblut et al. 2017), needs and motivations in ICU physicians, provides an explanation for their decisions (Votta et al. 2001) and actions (Rosenblatt 2000) which will then guide their timely, personalised and targeted support (Braun and Berg 1994).

## Impact of Death and Dying on Doctors

Several studies (Brooks et al. 2017a, 2017b, Çobanoğlu and Algıer 2004, Hamric and Blackhall 2007, Henrich et al. 2016, Trankle 2014) report that caring for dying patients cause moral, emotional and psychological distress and even disagreements amongst ICU physicians. These studies lend weight to the notion that caring for the dying in the ICU has wide ranging effects upon physicians. Concurrently, evidence of adaptations made to thinking, preferences, decision-making and conduct suggests that facing death and dying influences the beliefs, values and principles contained within each ring, ultimately impacting how the physician perceives their selfhood. For example, in a systematic scoping review of physicians caring for terminally ill children (Ngiam et al. 2021), physicians may struggle to rationalise harsh realities of their patient’s situation with their religious beliefs which could potentially lead to a stronger or weaker belief in the afterlife, causing their Innate Ring to shift as their spiritual views change. Due to the interrelatedness of the rings, such a change could cause a ripple effect in the Individual, Relational and Societal Rings, and potentiallya drastic change to their personhood.

## Catalysts

Experiences that instigate changes to a physician’s personhood due to ethical, moral or legal challenges to prevailing beliefs, values and principles contained within their Innate, Individual, Relational and/or Societal Rings are referred to as “catalysts”. For example, an ICU physician who holds strongly to the sanctity of life by virtue of their religious principles (Innate Ring) may face immense dissonance when confronted with their professional duty to withhold or withdraw life-sustaining treatment deemed clinically futile (Societal Ring).

In order to visualise how events surrounding a patient’s dying may serve as a catalyst inciting conflicts within the ICU physician, we consider the scenario of Physician A:

*Physician A is a respiratory physician who first diagnosed Patient X, a 75 year old man, with chronic pulmonary disease from smoking. About 6 months ago, Physician A diagnosed Patient X with Stage 4 metastatic lung cancer and referred him to an oncologist. Given his long association with Patient X, Physician A continued to review him. About a month ago, Patient X was told his cancer was progressing despite chemotherapy.*

*Patient X had sought Physician A’s advice on how to proceed, knowing that his treatment options were limited. Patient X then explained that he had found the chemotherapy very debilitating and was keen to stop all treatment and wanted to focus on maximising the quality of his remaining life. He had come to seek Physician A’s advice on this matter. He agreed for a Home Hospice Care referral and told Physician A that he wanted to spend his remaining days away from the hospital and in the company of his three children who lived abroad. He was looking forward to seeing his first grandson born to his eldest son. Physician A helped Patient X complete an Advanced Care Plan (ACP) and informed the oncologist of the patient’s desire to stop treatment.*

*Unfortunately, the next day, the borders of the country where Patient X’s children were living were closed due to efforts to curb the spread of COVID-19, leaving his two sons unable to secure outbound flights home. Fortunately, Patient X’s youngest daughter who was on a sailing holiday in Europe was already on-route and arrived just before the borders of Patient X’s country were sealed. She did however have to serve a two-week self-isolation period in a local government facility. Unfortunately, she tested positive for COVID-19 and remained at the facility for 38 days.*

*A week after she completed her self-isolation, Patient X became ill and was taken to hospital with a severe case of pneumonia. After Patient X desaturated in the general ward, he was transferred to the ICU. Physician A broached the topic of Patient X’s ACP with his daughter and explained that, although he was in the ICU, he would not be mechanically ventilated should he deteriorate further. In that moment, Patient X’s daughter pleaded with Physician A to “do everything and anything possible” to save her father. The daughter refused to acknowledge Patient X’s ACP since it had been made before the COVID-19 restrictions were put in place and done in the belief that he could at least see his grandson. Patient X’s daughter threatened legal action to have the ACP set aside arguing that it had been made without due consideration of the impact of the COVID-19 restrictions. Furthermore, she argued that now that the travel limitations had eased, a time-trial of ventilation should be allowed to buy time for her brothers to visit.*

**Fig. 2:**
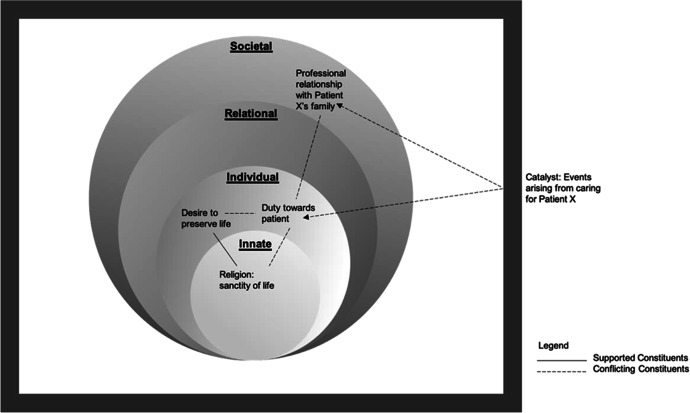
Catalyst interacting with Physician A’s Rings. Conflicting and supporting constituents that interact and affect Physician A’s Rings

Here, Patient X’s situation is considered a catalyst that brings forth numerous conflicts for Physician A. Firstly, Physician A recognises that whilst Patient X had made the ACP before the COVID-19 travel restrictions, he acknowledges that it was nonetheless a prior arrangement made (Societal Ring). In addition, he feels conflicted as he understands that Patient X would want to be able to see his children and grandson before he dies (Individual Ring). Furthermore, the ability of Physician A to address these conflicts are compromised by his own staunch religious beliefs in the sanctity of life (Innate Ring). The sheer incongruity between the beliefs, values and principles he upholds in the various Rings of Personhood leaves Physician A feeling deeply unsettled (Fig. 2).

## Internal Constituents and External Factors

Understanding the internal constituents and external factors at play is crucial to understanding how Physician A may address this catalyst and its resultant conflicts.

To clarify, internal constituents include Physician A’s beliefs, values, principles and moral considerations in addition to the roles, responsibilities and duties that may arise from his relational, social and professional affiliations. These are already encapsulated in the four rings of the RToP.

External factors, on the other hand, are contextual factors which throw the status quo within and between the rings into disarray. They remain outside of Physician A’s locus of control. Such external factors can be very real, including a poor working environment, poor intercollegial support and the lack of timely, personalised, accessible support at the institutional level, and the lack of rotations or roles outside of their ICU clinical duties to recuperate, reflect, process their ICU experiences and receive support if required(Kuek et al. 2020).

In this instance, out of the ordinary circumstances have been brought on by the COVID-19 crisis. More specifically, external factors immediately impacting the physician’s internal constituents would be the resource limitations posed by the outbreak and the pursuant triaging of ICU beds. Within the context of triaging in the pandemic, Patient X would not be a candidate for ventilatory support in ICU. Whilst such considerations are outside the locus of influence for the physician, they will nonetheless impact the physician keenly as they bear down on his internal constituents and problematise prior standards and norms by offering unprecedented scenarios with its own urgent set of needs and requirements. With different internal constituents affected and different external factors at large, it is clear that catalysts may have different effects on different people at different time points.

## Addressing Internal Constituents

A physician may address conflicts between internal constituents through prioritisation and/or reframing.

### Prioritisation

When faced with a catalyst that leads to conflict between constituent parts (beliefs, values and/or principles) within and between rings, physicians may choose to prioritise and place greater importance on one constituent over another. For example, Physician A may resist initiation of ventilatory support for Patient X, despite his daughter’s wishes, based on the belief and professional stance that patients should have autonomy over their own treatment.

### Reframing

Physician A could reframe competing constituents by altering his perspective of the situation at hand and viewing the decision to withhold ICU interventions as respecting the patient’s wishes. This would allow Physician A to meet his obligation to reduce suffering and, in the likelihood that the patient will not regain consciousness to see his children, abide by the ethical principle of beneficence as ICU treatment would be futile.

## Dyssynchrony

In prioritising one set of beliefs, values and principles over another and reframing perspectives, the physician may however find that the “solution” achieved is still in conflict with other constituents, particularly those contained in other rings. Whilst the different rings may offer mutual support for one another, this may not be the case if conflicts persist and are only partially resolved or poorly resolved. Conflicts that continue to persist between rings are referred to as “dyssynchrony” and may result in sharp and prolonged moral, spiritual, emotional and psychological distress. For the ICU physician, such dyssynchrony may bear severe repercussions on their decision-making and functioning as efficacious healthcare professionals with vulnerable and dependent patients under their care.

**Fig. 3:**
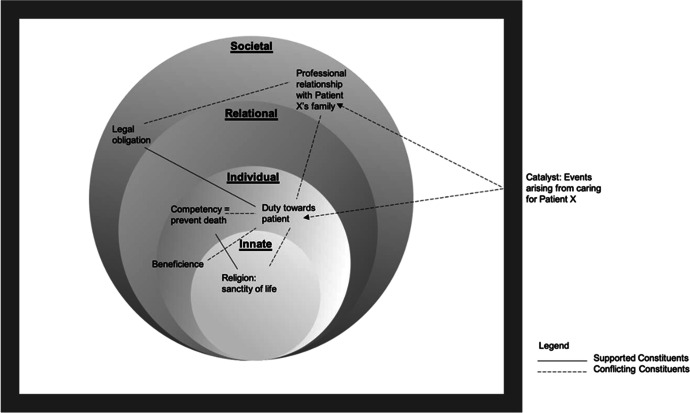
“Dyssynchrony” in Physician A’s Rings of Personhood. Dyssynchrony due to supporting and conflicting constituents within Physician A’s Rings

In prioritising the specific beliefs, values and principles that arise from his professional role and responsibilities, Physician A experiences a greater chasm within his Innate Ring which upholds the sanctity of life and his Societal Ring which sees him desiring to dutifully support his patient’s family as best he can. As such, left unchecked, poor resolution of these issues will lead to dyssynchrony between the rings (Fig. 3). It is also evident that in addressing these conflicts, the physician must be mindful of the longitudinal and holistic effects of finding such solutions. It is further suggested that addressing or resolving dyssynchrony is key to supporting these ICU physicians.

Indeed, in discussing the prioritisation and reframing of internal constituents, the impact of external factors become apparent. A tired, emotionally drained physician at the end of a busy week at work confronted with a difficult issue involving persons that they can “relate to” will likely influence their ability to adequately address these conflicts and temper or resolve the dyssynchrony which may follow.

**Fig. 4:**
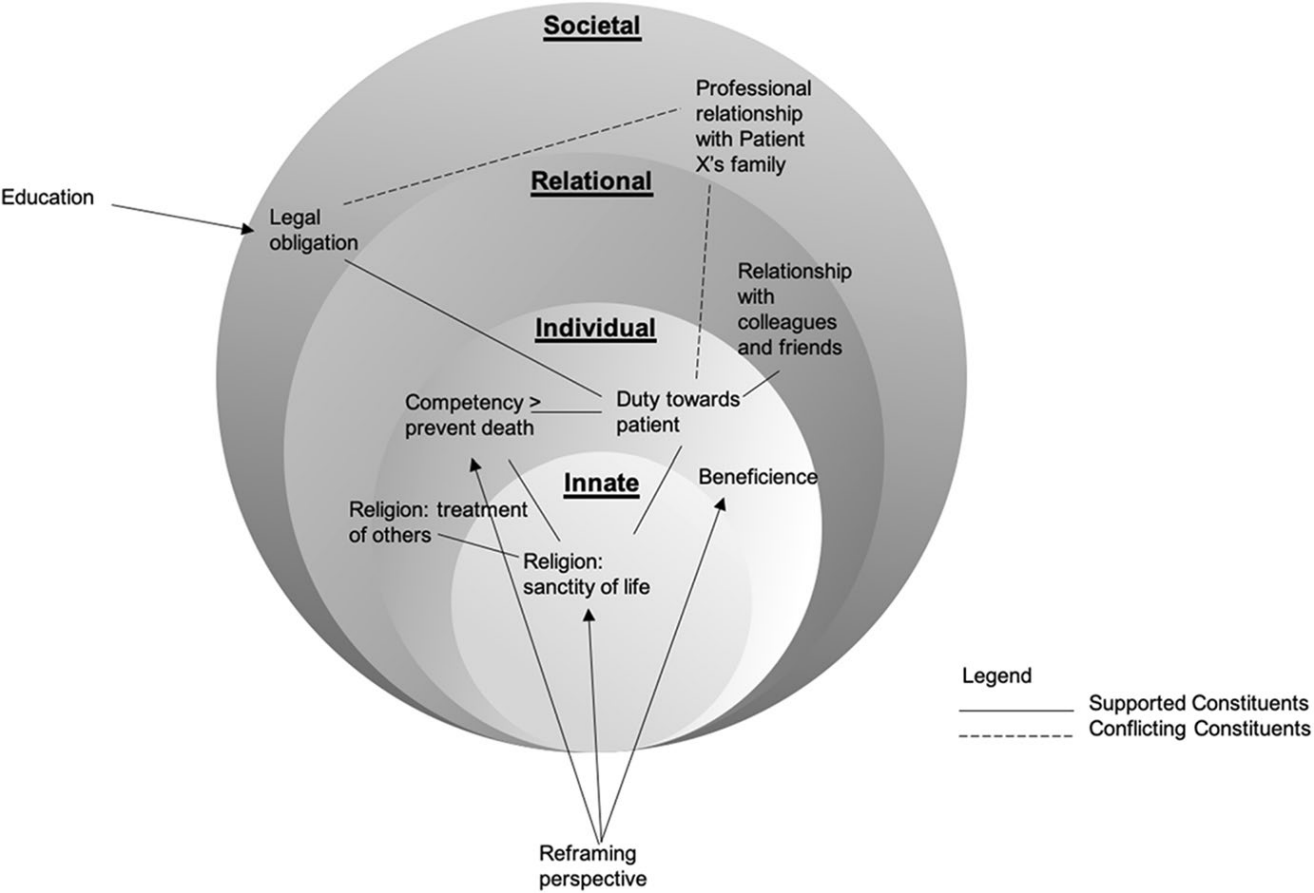
Physician A’s movement from dyssynchrony to synchrony. Movement from dyssynchrony to synchronny within Physician A’s Rings

Fortunately, a good support system surrounds Physician A. In discussing the situation with a colleague and friend, Physician A realises that disregarding patient autonomy has great ethical and legal implications upon his professional and personal beliefs, values and principles. He reasons, and is supported by his colleague and friend, to see that whilst he shared a bond with the patient, this bond may have clouded his decision-making process. Through an open conversation with his religious leaders and deeply reflecting on Patient X’s wishes, Physician A acknowledges that his personal conviction to sustain and prolong life in all circumstances may be detrimental to his patient’s quality of life. As such, he knows that he has to honour the ACP. Having arrived at a firm decision and resolving the conflict within (Fig. 4), Physician A moves towards a state of synchrony.

Here, a positive influence of external factors is evident—that there are clear benefits to having timely, personalised and holistic clinical, professional and individualised collegial support. A nurturing working environment, opportunities to seek support, and time to reflect was key to supporting Physician A in finding synchrony between his Rings of Personhood.

## Buffers

It is also likely that similar situations in the future will have less of a detrimental impact upon Physician A. Indeed, an ICU physician who is fortified with prior experience and well supported by colleagues and members of their Relational Ring will be better able to address catalysts even before they create conflict or dyssynchrony. Termed as “buffers”, we suggest that experience, access to support, time to process, reflect and inculcate lessons learnt in a supportive working environment are in part the basis for the physician’s ability to address catalysts, resolve conflicts and develop resilience against them. Notably, buffers not only emerge from external factors but also build upon internal constituents of the rings and may be present in all rings.

## Reconceiving the RToP

The presence of catalysts, conflicts, prioritisation and reframing, external factors and internal constituents, buffering, synchrony and dyssynchrony highlight that concepts of personhood evolve and require review.

**Fig. 5:**
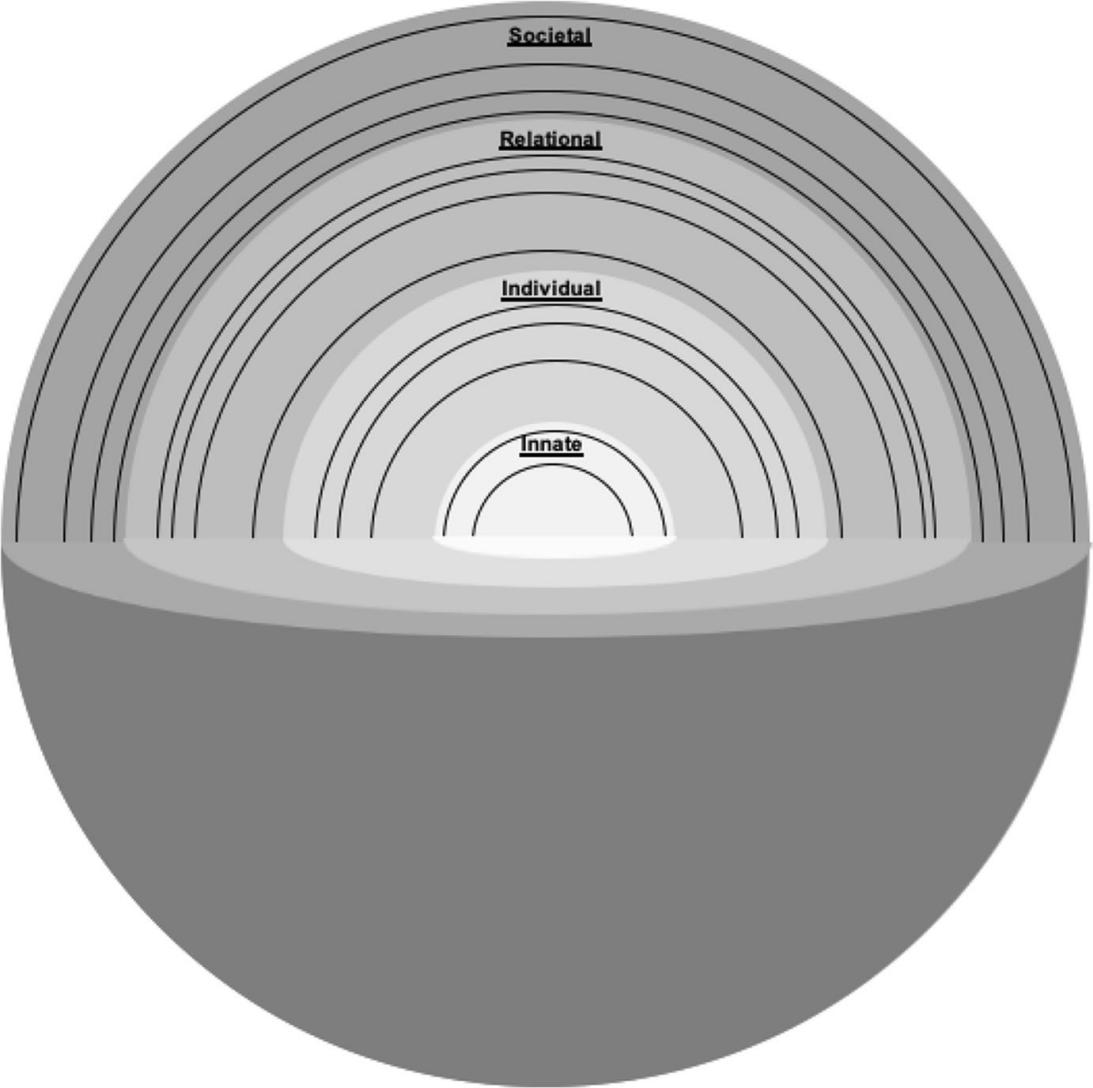
Spheres of personhood. Moving from rings to the spheres of personhood

To begin, the data suggests that concepts of personhood are not solely concerned with sustaining concepts of “what makes you, you” within the settings of dementia and palliation sedation (Buron 2008; Radha Krishna et al. 2015; Radha Krishna and Alsuwaigh 2015). Rather, it involves the maintaining of a coherent and authentic representation of one’s selfhood in “peace time” and in precarious climates where incoming catalysts may incite conflict and dyssynchrony between the various aspects of an individual’s concept of personhood, in turn, is determined by the presence and quality of buffers. The use of spheres here serve to underline the need to consider the various critical aspects of personhood as identified in this paper (Fig. 5).

These layered spheres reiterate the intertwined nature of the previous rings by maintaining a porous border between it and other layers. These porous borders explain the occurrence of the “ripple effect” when conflict arises between the rings/spheres and predispose dyssynchrony. Concurrently, these porous boundaries facilitate support for the rings/spheres in conflict as seen when Physician A’s colleagues and religious leaders who occupy a place in his Societal Ring/Sphere help address the dyssynchrony between his religious beliefs (Innate Ring/Sphere) and professional responsibilities (Societal and Individual Ring/Spheres). The porous borders may see the outer layers of the Relational Ring/Sphere, in some circumstances, shift into the Societal Ring/Sphere and vice versa.

Changes in the ring/spheres due to conflict will see elements of internal constituents move further away from the sphere’s centre as a result of being accorded a lower level of importance or ranking. These elements may also reduce in size if associated buffers are attenuated.

## Conclusion

Conceiving the effects of caring for dying patients in ICU from evidence-based data (Kuek et al. 2020) through the lens of the RToP emphasises that the impact of working in the ICU has wide ranging effects upon physicians. Psychological, emotional, academic, research, administrative, personal and professional matters may come together to impact the Innate, Individual, Relational and Societal Rings of their RToP, eventually shifting their needs, values, thinking, motivations and actions with ripple effects.

Thus, from a practical perspective, acknowledging the effects of caring for patients in ICU from a holistic perspective merely foregrounds the need for a concerted effort to comprehensively support clinicians. This framework provides a means of guiding support for ICU physicians at a timely, personalised and targeted manner on two levels - on a personal level, to educate individuals in addressing any conflicts between their internal constituents through prioritisation and reframing, and on an institutional level, to aid host organisations in establishing supportive measures, such as role rotations, timely conflict management interventions, avenues for physicians to share their emotions and experiences. These will help to reduce instances of dyssynchrony, address negative external factors, enhance buffers and ensure a nurturing work environment for ICU physicians.

Understanding the impact of dyssynchrony as a source of internal strife stemming from poor support for ICU physicians also underscores the need to determine how to better assess their needs. Here, the spheres of the RToP may serve as the basis for a longitudinal assessment tool. Such assessments need to be supported by use of portfolios and individualised mentoring processes that provide personalised, appropriate, specific, timely, accessible and long-term support. This would be especially useful for junior physicians in ICU.

The provision of such care for the ICU physician is particularly pertinent whilst the COVID-19 situation rages on and healthcare professionals continue to fight seemingly inextinguishable fires. It is our hope that this paper will help institutions and physicians alike recognise how this striving can take its toll on the firefighter and set out to better equip and protect them from the fire itself.

## Data Availability

All data generated or analysed during this study are included in this published article and its supplementary information files.

## Abbreviations

COVID-19: Coronavirus disease 2019
ICU: Intensive Care Unit
RToP: Ring Theory of Personhood
ACP: Advance Care Plan
